# Hidden Hunger in Pediatric Obesity: Redefining Malnutrition Through Macronutrient Quality and Micronutrient Deficiency

**DOI:** 10.3390/nu17223601

**Published:** 2025-11-18

**Authors:** Vanessa Nadia Dargenio, Nicoletta Sgarro, Giovanni La Grasta, Martina Begucci, Stefania Paola Castellaneta, Costantino Dargenio, Leonardo Paulucci, Ruggiero Francavilla, Fernanda Cristofori

**Affiliations:** Interdisciplinary Department of Medicine, Pediatric Section, Children’s Hospital ‘Giovanni XXIII’, University of Bari “Aldo Moro”, 70126 Bari, Italy; vanessa.dargenio@unifg.it (V.N.D.); nicolettasgarro@gmail.com (N.S.); g.lagrasta94@gmail.com (G.L.G.); martinabegucci@gmail.com (M.B.); castellanetas@gmail.com (S.P.C.); costy.dargenio@gmail.com (C.D.); leonardo.paulucci92@gmail.com (L.P.); fernandacristofori@gmail.com (F.C.)

**Keywords:** obesity, malnutrition, micronutrients, macronutrients, deficiency, nutrition, children

## Abstract

**Background:** Pediatric obesity exemplifies the paradox of energy excess coexisting with nutritional inadequacy. Despite high caloric intake, children with obesity often display deficiencies in essential macro- and micronutrients that impair growth, metabolic regulation, and long-term health. This review critically examines the mechanisms underlying malnutrition in pediatric obesity, emphasizing the interplay between dietary quality, inflammation, microbiota alterations, and biomarker profiles, and identifies research gaps limiting precision nutrition approaches. **Methods:** A comprehensive narrative review of studies addressing macro- and micronutrient intake, metabolic and inflammatory biomarkers, and gut microbiota–host interactions in pediatric obesity was conducted. Evidence from both clinical and experimental models was integrated to evaluate mechanistic pathways, diagnostic criteria, and preventive strategies. **Results:** Obesity-related malnutrition arises from poor dietary quality, systemic inflammation, and microbiota dysbiosis, leading to impaired nutrient utilization and metabolic dysfunction. Deficiencies in vitamin D, calcium, iron, magnesium, and B vitamins are common and often coexist with macronutrient imbalances. Diets rich in saturated fats and refined carbohydrates exacerbate inflammation and metabolic risk, whereas plant-based proteins, unsaturated fats, and fiber support metabolic resilience. Precision nutrition and biomarker-guided monitoring show promise but require validation in pediatric cohorts. Evidence on microbiota modulation and nutrient–gene interactions remains inconsistent, reflecting methodological heterogeneity. **Conclusions:** Malnutrition in pediatric obesity should be recognized as a distinct clinical phenotype characterized by qualitative nutrient deficiency within a state of energy surplus. Addressing this paradox demands harmonized diagnostic criteria, longitudinal biomarker surveillance, and individualized dietary strategies informed by genetics and microbiome profiling. Multilevel interventions, linking clinical practice, policy, and food system reform, are essential to prevent lifelong metabolic complications and promote healthy growth trajectories.

## 1. Introduction

Malnutrition in pediatric populations remains a major global health challenge, encompassing both undernutrition and overnutrition, each with significant implications for growth, development, and long-term health. The World Health Organization (WHO) defines malnutrition as “deficiencies, excesses, or imbalances in a person’s intake of energy and/or nutrients,” including undernutrition (wasting, stunting, underweight) and overweight/obesity [1]. Globally, malnutrition contributes to nearly 45% of deaths among children under five years of age, with undernutrition being the leading cause of mortality in low- and middle-income countries [2]. Conversely, the rising prevalence of childhood obesity, particularly in high-income settings, illustrates a paradoxical form of malnutrition characterized by excessive caloric intake coupled with qualitative nutrient deficiencies [3].

The coexistence of undernutrition and obesity within the same population, household, or even individual, known as the dual burden of malnutrition, poses a substantial challenge to global health systems [4]. Studies in Malaysia and Zimbabwe, for instance, have reported concurrent high rates of stunting and obesity in children, largely driven by urbanization, dietary transition, and socioeconomic disparity [4]. Such findings emphasize the urgent need for integrated strategies addressing both extremes of the nutritional spectrum. Undernutrition manifests as wasting [BMI > 3 SDs below the age-specific mean], stunting (height-for-age z-score < −2 SDs from the WHO median), and underweight (low weight-for-age). According to recent WHO estimates, 149 million children under five are stunted, while 45 million experiences wasting [5]. Beyond impaired physical growth, undernutrition compromises cognition, immunity, and metabolic function, perpetuating cycles of poverty and reduced productivity [6,7]. In developing regions, it is primarily driven by food insecurity, infections, and poor sanitation [8], whereas in high-income settings it often results from chronic diseases, malabsorption syndromes such as celiac disease (CD), and socioeconomic inequalities [9,10]. Approximately 25% of hospitalized children in the U.S. exhibit acute protein–energy malnutrition [11].

Over the past four decades, the prevalence of childhood obesity has tripled, affecting over 340 million children and adolescents globally [12]. Obesity, defined as excess body fat accumulation beyond physiological requirements, predisposes to diabetes, hypertension, dyslipidemia, and cancer [13]. Its etiology reflects a multifactorial interplay of environmental, behavioral, and genetic determinants. The global dietary shift toward energy-dense, nutrient-poor processed foods has produced a paradoxical form of malnutrition in which caloric surplus coexists with micronutrient deficiency [14]. Diets dominated by ultra-processed foods further promote insulin resistance, inflammation, and non-communicable diseases [15].

This double burden of malnutrition is most evident when obese children exhibit stunting or micronutrient deficiencies [16]. Obesity-associated dysbiosis of the gut microbiota exacerbates inflammation and disrupts nutrient absorption [17]. Intergenerational effects also play a role: in Peru, for instance, children of overweight mothers have a higher risk of developing obesity [18]. Beyond excessive intake, the mechanisms linking obesity and malnutrition involve chronic low-grade inflammation. Adipose tissue functions as an endocrine organ, releasing cytokines such as TNF-α, IL-1β, and IL-6 that disrupt metabolic homeostasis and hypothalamic energy regulation. This inflammatory milieu alters nutrient utilization, increases metabolic demands, and contributes to sarcopenia, the loss of skeletal muscle mass and function. The coexistence of obesity and sarcopenia defines sarcopenic obesity, a severe yet underrecognized phenotype associated with poor outcomes. Macronutrient quality and balance critically determine metabolic health [19]. Competing theoretical models illustrate this complexity: the Carbohydrate–Insulin Model (CIM) emphasizes high-glycemic carbohydrates; the Energy Balance Model (EBM) highlights caloric surplus from processed foods; the Fructose Survival Hypothesis (FSH) focuses on fructose metabolism [20]; and the Protein Leverage Hypothesis (PLH) suggests that inadequate protein density drives compensatory overeating [21]. Together, these frameworks underscore the need to move beyond calorie counting toward understanding macronutrient interactions and quality.

A modern approach to pediatric nutrition must integrate both quantitative and qualitative assessments of macro- and micronutrients [19], since optimal intake is essential for growth and development. Biomarkers have emerged as indispensable tools for early detection of malnutrition, offering objective indices that precede clinical signs. They include cytokines, interleukins, hormones, and markers of oxidative stress that reflect metabolic and inflammatory status.

This narrative review synthesizes current evidence on malnutrition in obesity, examining underlying mechanisms, clinical consequences, and the interplay between macro- and micronutrient imbalance, with special emphasis on biomarkers for early diagnosis and targeted management. To critically appraise this complex phenomenon, the review will first dissect the established and putative biological pathways, in particular dietary quality, chronic inflammation, and gut microbiota interactions, that drive nutrient deficiencies. It will then detail the specific macro- and micronutrient imbalances and their clinical sequelae, concluding with an evaluation of emerging diagnostic and therapeutic strategies. A comprehensive literature search was conducted in the PubMed, Scopus, and Web of Science databases for studies published between 2000 and 2025. The search combined terms related to “malnutrition,” “childhood obesity,” “undernutrition,” “biomarkers,” and “pediatric nutrition.” The search was restricted to full-text articles in English involving human pediatric populations (0–18 years) and published in recent years. Following screening for relevance by two researchers (V.N.D. and N.S.), the identified literature was critically synthesized in a narrative format, focusing on mechanistic insights, clinical implications and emerging biomarker applications. As a narrative review, no formal protocol was registered, and no quantitative meta-analysis was per-formed.

## 2. Definitions and Guidelines for Pediatric Malnutrition

Historically, pediatric malnutrition was often suspected based solely on clinical appearance, without standardized or objective diagnostic tools. The focus has now shifted toward establishing evidence-based and universally applicable diagnostic criteria that ensure consistency across healthcare settings. Previously, the absence of a unified definition encompassing both developing and developed countries led to substantial variability in reported prevalence, ranging from 6% to 51%, and to inconsistent screening practices across institutions. To address this gap, an interdisciplinary task force from the American Society for Parenteral and Enteral Nutrition (ASPEN) reviewed the available evidence and proposed a consensus definition of pediatric malnutrition. ASPEN defines malnutrition as “an imbalance between nutrient requirements and intake, resulting in cumulative deficits of energy, protein, or micronutrients that negatively affect growth, development, and clinical outcomes” [22].

This marked a major conceptual shift, from a weight-based assessment to a multidimensional framework incorporating five core domains: (a) anthropometrics (Z-scores, BMI-for-age), (b) chronicity (acute vs. chronic malnutrition), (c) etiology (disease-related vs. environmental causes), (d) functional outcomes, such as immune impairment or delayed wound healing, and the recognition that multiple factors often coexist.

Following this redefinition, a joint ASPEN–Academy of Nutrition and Dietetics task force developed standardized diagnostic indicators for children aged 1 month to 18 years. Two main categories were proposed: (a) single data-point indicators, which can independently diagnose malnutrition; and (b) comparative indicators, which require the evaluation of two or more data points over time (see Table 1 and Table 2).

The consensus recommends using WHO growth charts for children under 2 years of age and CDC growth charts for those older than 2 years. The key anthropometric indicators include: (a) Weight-for-length (for children <2 years), (b) BMI-for-age (for children >2 years), (c) Length/height-for-age, and (d) Mid-upper arm circumference (MUAC).

Any of these indicators may be sufficient to establish a diagnosis. However, to determine malnutrition severity, at least two of the following four dynamic indicators are required: (a) Reduced weight-gain velocity (<2 years), (b) Weight loss (>2 years), (c) Deceleration in weight-for-length or BMI-for-age Z-score, and (d) Inadequate nutrient intake [24].

These criteria represent an important initial framework to standardize the diagnosis and documentation of pediatric malnutrition. Nevertheless, current evidence remains limited regarding their diagnostic accuracy, including sensitivity, specificity, and predictive validity across populations [22].

To facilitate future validation, clinicians are strongly encouraged to systematically record anthropometric and nutritional parameters in electronic medical records, enabling large-scale data collection, auditing, and refinement of diagnostic indicators.

### Assessing Nutritional Status Using Centile and Z-Score Charts

The assessment of nutritional status in children traditionally relies on plotting anthropometric parameters on centile (percentile) growth charts, which compare an individual’s growth pattern with that of a reference population [25]. Percentiles indicate a child’s relative position within the population distribution, allowing clinicians to identify deviations from expected growth trajectories over time. However, these charts have inherent limitations: while they depict relative position, they do not quantify the magnitude of deviation from the mean, thus limiting sensitivity in detecting subtle or progressive nutritional deficits [26].

To overcome these limitations, Z-score (standard deviation score) charts have become the preferred tool for evaluating pediatric growth and nutritional status. Z-scores express anthropometric measurements as the number of standard deviations above or below the reference mean, providing a precise and continuous numerical representation [27]. A Z-score of 0 corresponds to the median (50th percentile), whereas values between −1 and +1 indicate normal growth. Values below −2 denote moderate malnutrition, and those below −3 indicate severe malnutrition, roughly corresponding to the 3rd percentile [24].

Unlike percentile charts, Z-scores enable detection of extreme values and facilitate longitudinal monitoring, even in children with atypical or very low growth trajectories. This precision makes them particularly valuable for evaluating the efficacy of nutritional interventions and for comparing growth data across populations and studies [28]. Consequently, Z-score-based growth standards are now considered the gold standard for diagnosing, classifying, and tracking pediatric malnutrition.

## 3. Screening and Diagnosis in Clinical Practice

Nutritional screening in pediatric populations represents a cornerstone of clinical practice and the first line of defense against the adverse effects of malnutrition [29]. Early identification of nutritional deficits allows for timely intervention, which can profoundly influence disease progression and clinical outcomes [30]. Given the diversity of healthcare settings, ranging from tertiary hospitals to resource-limited community clinics, screening approaches must be adapted to local operational capacities.

In hospitalized children, the ASPEN recommends performing nutritional screening within the first 24 h of admission [31]. Early assessment is critical, as delayed recognition of malnutrition is associated with prolonged hospitalization, increased infection risk, and poorer recovery trajectories. Evidence demonstrates that prompt nutritional support mitigates disease-related malnutrition, particularly among critically ill and chronically affected children [31].

In community settings, especially in low- and middle-income countries, screening tools must be simple, scalable, and independent of laboratory infrastructure. Anthropometric indicators such as mid-upper arm circumference (MUAC) and weight-for-height Z-scores are widely employed due to their cost-effectiveness, reliability, and ease of implementation. These measures enable rapid identification of at-risk children by frontline health workers, facilitating timely referral and nutritional intervention.

Several validated screening instruments have been developed to standardize nutritional risk assessment in pediatric care.

(a) The STRONGkids tool demonstrates high validity and reproducibility in detecting disease-related malnutrition by integrating clinical and dietary risk factors, making it suitable for both inpatient and outpatient settings.
(b) The Screening Tool for the Assessment of Malnutrition in Pediatrics (STAMP) has shown particular accuracy in critically ill children, where higher scores correlate with extended hospital stays and increased mortality.
(c) The Pediatric Yorkhill Malnutrition Score (PYMS) exhibits strong sensitivity and specificity in specialized populations, such as pediatric oncology patients, facilitating early identification of those requiring targeted nutritional support.

Timing is a crucial consideration. Screening should be conducted immediately upon hospital admission, especially in high-risk conditions such as congenital heart disease, cancer, and chronic gastrointestinal disorders. For patients with extended stays, periodic rescreening (at least weekly) is recommended to identify hospital-acquired or evolving malnutrition before clinical deterioration occurs [32]. To translate screening into action, a positive score on tools like STRONGkids or STAMP should automatically trigger a comprehensive nutritional assessment by a registered dietitian and the initiation of a nutritional care plan. Furthermore, in the context of pediatric obesity, nutritional screening must be considered a continuous process. An initial assessment upon diagnosis should be followed by annual re-evaluations, or more frequently if the child’s BMI trajectory is rapidly increasing or if they show signs of metabolic complications, to monitor for the development of hospital-acquired or progressive malnutrition.

Optimal management of pediatric malnutrition requires a multidisciplinary approach, integrating pediatricians, dietitians, nurses, and allied health professionals [33]. While existing guidelines provide a robust framework, clinical strategies must be individualized, accounting for each child’s underlying condition, clinical status, and environmental context. Proactive and systematic screening remains pivotal for preventing long-term sequelae and optimizing health outcomes in pediatric populations.

## 4. Biological Mechanisms of Malnutrition in Obesity: Nutrient Deficiencies and Metabolic Dysfunction

The malnutrition paradox in obesity is not a passive state of inadequate intake but an active pathological process driven by the interplay of diet quality, systemic inflammation, and metabolic dysfunction [34]. The sequence of events often begins with the consumption of modern hypercaloric diets, dominated by refined carbohydrates, industrial fats, and low-quality proteins. It is well-established that this dietary pattern directly displaces nutrient-dense foods, lowering micronutrient intake, predisposing individuals to cardiometabolic disorders [28,34,35]. Concurrently, obesity-related physiological adaptations, including increased blood volume, adipose tissue expansion, and organ remodeling, modify nutrient distribution and bioavailability, producing functional deficiencies even when intake appears adequate [36]. Furthermore, strong evidence supports that such diets promote adipose tissue expansion and a chronic state of low-grade inflammation. This inflammatory milieu, in turn, plausibly contributes to nutrient deficiencies through several mechanisms: elevated hepcidin impairing iron absorption, sequestration of fat-soluble vitamins like vitamin D in adipose tissue, and increased oxidative stress depleting antioxidant reserves [37]. Elevated leptin concentrations, often accompanied by leptin resistance, disrupt energy homeostasis and worsen metabolic dysfunction [38]. Simultaneously, deficiencies in micronutrients such as zinc, magnesium, and vitamin D impair insulin signaling, thereby intensifying insulin resistance and type 2 diabetes. Dysfunctional adipose tissue further releases pro-inflammatory cytokines, reinforcing systemic inflammation and exacerbating nutrient metabolism abnormalities [39].

A more speculative, yet compelling, hypothesis implicates obesity-related gut dysbiosis in exacerbating this cycle by altering nutrient synthesis and absorption, and by increasing gut permeability, which further fuels systemic inflammation [40]. Thus, obesity-related malnutrition arises from a synergistic interplay of dietary composition, inflammatory signals, hormonal dysregulation, and altered nutrient distribution [34]. Together, these mechanisms lead to reduced levels of vitamins, iron, calcium, and magnesium, micronutrients that may serve as potential biomarkers of malnutrition in obesity.

### 4.1. Clinical Significance of Malnutrition in Obesity

The clinical consequences of malnutrition in obesity extend far beyond micronutrient imbalance, amplifying a wide range of comorbidities (Table 3).

Under conditions of nutrient deficiency, micronutrients are preferentially allocated to vital organs, leaving peripheral tissues metabolically compromised. For instance, vitamin K is prioritized for hepatic coagulation functions at the expense of bone metabolism, contributing to reduced bone mineral density and increased fracture risk [42]. Similarly, deficiencies in vitamin D and magnesium impair glucose metabolism and pancreatic β-cell function, exacerbating insulin resistance and promoting the onset of type 2 diabetes [42]. Malnutrition also compromises skeletal muscle anabolism, predisposing individuals to sarcopenic obesity, in which excess adiposity coexists with loss of lean mass and reduced functional capacity [42]. Additional consequences include cognitive dysfunction, reproductive disturbances, and adverse metabolic outcomes, reflecting the systemic impact of nutrient deprivation within an inflammatory and obesogenic environment [42].

In pediatric populations, this burden is particularly concerning. An estimated 78.4% of obese children in the United States exhibit at least one nutritional deficiency, which increases the risk of early-onset dyslipidemia, hypertension, and insulin resistance [44]. These findings underscore that obesity-related malnutrition is not a marginal issue but a pervasive clinical entity with significant short- and long-term implications.

From a health system perspective, malnutrition in obesity also increases healthcare utilization and mortality risk. Obese patients represent nearly one-third of intensive care unit (ICU) admissions, and outcomes are significantly worse when malnutrition coexists with obesity [44]. This paradox, caloric overconsumption concurrent with essential nutrient deficiency, reinforces the need for early recognition and individualized nutritional management across all levels of care.

**Figure 1:**
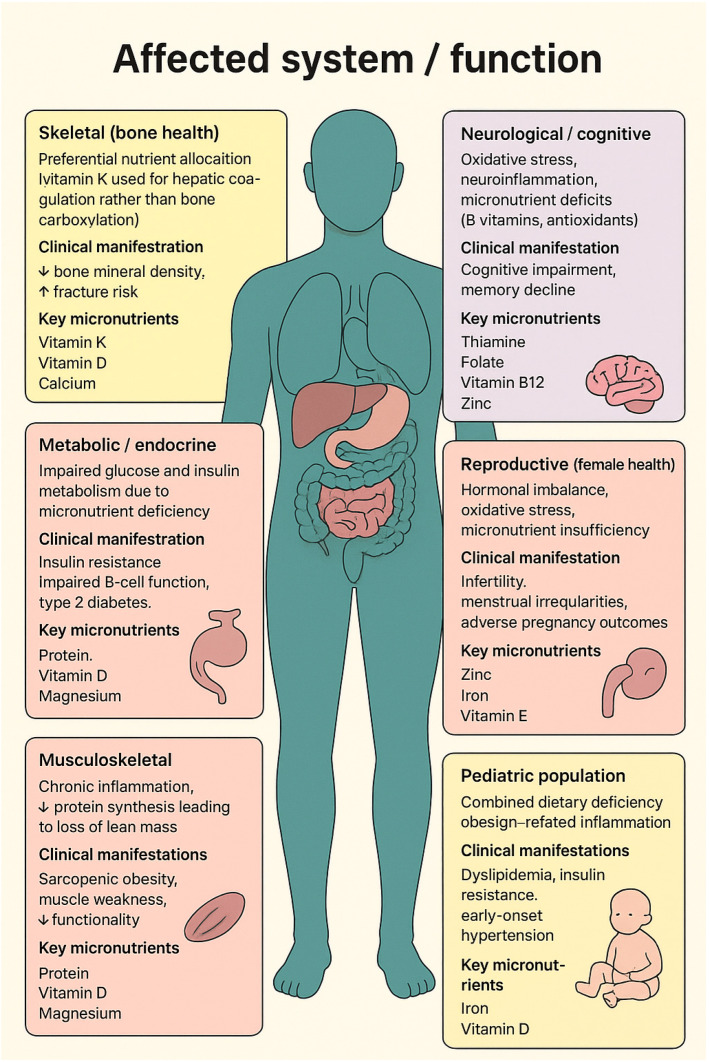
The affected organs, clinical manifestations, and key nutrients involved in obesity-related malnutrition. ⭡ increase, ⭣ reduce.

The main underlying mechanisms linking micronutrient deficiencies to systemic dysfunctions are summarized in Figure 1.

#### 4.1.1. Single Nutrient Models of Obesity

The CIM posits that high-glycemic index (GI) carbohydrate intake is a major driver of obesity. Consumption of refined carbohydrates elevates circulating insulin levels, increases the insulin-to-glucagon ratio, and shifts the gastric inhibitory polypeptide (GIP)–glucagon-like peptide-1 (GLP-1) balance toward lipogenesis [34]. Elevated insulin suppresses lipolysis, enhances hepatic de novo lipogenesis, and accelerates glucose uptake, creating a state of “internal starvation” in which peripheral tissues signal energy scarcity despite caloric abundance. This metabolic imbalance promotes hunger, hyperphagia, and sustained positive energy balance [45].

In contrast, the EBM attributes obesity to chronic energy surplus resulting from excessive caloric intake relative to expenditure. This model emphasizes the consumption of energy-dense foods, particularly those rich in fats and sugars, as the main determinant of sustained adiposity [46]. Because fat provides more than twice the caloric density of carbohydrates or proteins, excessive fat intake plays a predominant role in the development of obesity [47]. Although some evidence suggests a decline in basal metabolic rate may contribute to weight gain, caloric excess remains the principal determinant [48].

While both models acknowledge the obesogenic potential of dietary sugars, they diverge regarding the underlying mechanisms and primary nutrient drivers. The EBM locates the defect mainly within the central nervous system, where hyperpalatable foods override satiety signaling and enhance reward-driven eating. The CIM, on the other hand, identifies the defect within peripheral metabolic pathways, where altered substrate partitioning directs excess energy toward fat storage. While animal models support the CIM, rodents fed high-GI diets exhibit adipose accumulation preceding or occurring independently of increased caloric intake [49], the evidence in humans is more heterogeneous. Although some epidemiological studies corroborate these findings, large-scale interventional trials have sometimes yielded conflicting results, and the model’s primacy over the energy balance model remains a subject of debate [20,46]. The CIM is particularly challenging to test in isolation in free-living humans, and many studies rely on surrogate markers rather than direct measures of adiposity.

A complementary hypothesis, the FSH, implicates fructose, whether consumed as sucrose, high-fructose corn syrup (HFCS), or produced endogenously via the polyol pathway, as a unique metabolic driver of obesity [50]. Unlike glucose, fructose exhibits a low GI (~25) and elicits minimal insulin response. However, it strongly stimulates hepatic de novo lipogenesis, depletes intracellular ATP, and increases uric acid production, impairing mitochondrial function and promoting insulin resistance [51]. Chronic fructose exposure induces leptin resistance and persistent hunger, promoting adiposity despite cellular energy deficit. Epidemiological trends linking the introduction of HFCS in the 1970s with the global rise in obesity provide additional support for this model [52].

Collectively, the CIM, EBM, and FSH offer complementary yet competing frameworks for understanding obesity pathogenesis. They address the central question: Is obesity primarily driven by caloric excess, altered nutrient partitioning, or specific metabolic effects of sugars and fats? The EBM emphasizes caloric equivalence across macronutrients, whereas the CIM proposes that glucose-derived calories are more obesogenic than those from protein or fat. The FSH further refines this view, suggesting that fructose calories exert uniquely lipogenic and metabolically disruptive effects [53,54]. All these mechanisms are synthesized in Table 4.

#### 4.1.2. Insights from the Nutritional Geometry Framework (NGF)

The Nutritional Geometry Framework (NGF) offers a multidimensional approach to obesity, emphasizing how interactions among macronutrients shape metabolic outcomes. Central to this model is the PLH, which proposes that humans and animals tightly regulate protein intake and increase total energy consumption when dietary protein is diluted by fats or carbohydrates [65]. This compensatory hyperphagia promotes energy excess and adiposity, a mechanism particularly relevant to modern ultra-processed diets.

Experimental evidence supports this theory. In a landmark study, Solon-Biet et al. tested 25 diets in more than 700 mice and found that low-protein, high-carbohydrate (LPHC) diets increased food intake and fat mass but paradoxically improved cardiometabolic health and longevity, while high-protein, low-carbohydrate (HPLC) diets reduced lifespan despite less adiposity [66]. Similarly, Tordoff and Ellis demonstrated that maximal adiposity occurred in mice consuming balanced fat-to-carbohydrate ratios [67]. When analyzed through the NGF, these findings confirmed that macronutrient balance and carbohydrate quality, not calories alone, determine adiposity and metabolic risk. Subsequent studies refined this model by incorporating carbohydrate type and protein content. Wali et al. evaluated 33 isocaloric diets in 700 mice, showing that LPHC diets containing resistant starch improved metabolic health, while those containing glucose–fructose mixtures (equivalent to high-fructose corn syrup) produced the worst outcomes, including gut dysbiosis [68]. These findings demonstrate that the obesogenic potential of carbohydrates depends on both fat proportion and fiber content, highlighting the “LPHC paradox.” In high-fat diets, sugar-related effects diminish, and fat-induced insulin resistance predominates [56,69].

Together, the EBM, CIM, FSH, and NGF models provide complementary perspectives on obesity pathogenesis. The NGF integrates these frameworks, showing that obesity results not merely from caloric excess but from imbalanced macronutrient interactions, where protein dilution, carbohydrate type, and fat quality jointly influence energy intake, adiposity, and metabolic health. Future dietary strategies should therefore emphasize adequate protein intake, Reduction In Refined sugars (particularly HFCS), and optimization of fat sources, moving beyond calorie-based prescriptions toward a nutrient-quality paradigm.

### 4.2. Factors Contributing to Adolescent Obesity

#### 4.2.1. Diet Quality

Diet quality is a key determinant of adolescent nutritional status and obesity risk. It is commonly assessed using the Healthy Eating Index (HEI), derived from dietary recalls or food frequency questionnaires [70]. The National Health and Nutrition Examination Survey (NHANES) currently applies two 24 H recalls to calculate HEI scores, evaluating both overall diet quality and specific components such as saturated fats, added sugars, and sodium [70,71]. The HEI has evolved alongside the Dietary Guidelines for Americans (DGAs), with its most recent version, HEI-2015, comprising 13 components categorized as adequacy (foods to consume more of) and moderation (foods to limit) [72,73]. Scores range from 0 to 100, with higher values reflecting greater adherence to dietary recommendations.

Data from multiple NHANES cycles (1999–2012) reveal persistently low HEI scores among U.S. adolescents, which decline progressively with age [74]. The mean HEI-2015 score of 52.0 remains below optimal levels, indicating inadequate dietary quality. Although adolescents generally meet protein recommendations, their fatty acid profiles are suboptimal: the mean HEI subscore for fatty acids was only 3.7 out of 10, indicating excessive consumption of saturated relative to unsaturated fats [74].

Intake of fruits, vegetables, and whole grains remains markedly below recommendations [74,75,76,77,78]. Adolescents consume roughly half the recommended daily servings of fruits and vegetables and achieve an average score of only 1.32 out of 10 for whole grains [77]. As a result, dietary fiber has been identified as a nutrient of concern in the 2015–2020 DGAs. Although recent decades show a modest decline in “empty calorie” intake, added sugar consumption continues to exceed recommended limits, meeting only about 50% of the reduction target [74,79]. These findings highlight persistent gaps between current adolescent dietary patterns and national recommendations, emphasizing the need for comprehensive interventions that promote nutrient-dense food choices and improved dietary quality.

#### 4.2.2. Body Weight and Macronutrients

The regulation of body weight and energy metabolism has long been debated, with multiple theories explaining how macronutrients and physiological mechanisms influence obesity [80]. The glucostatic theory posits that short-term appetite regulation is governed by glucose availability in the brain, while the lipostatic model maintains that long-term body weight is stabilized by mechanisms preserving adipose mass.

Carbohydrate quality, defined by fiber content, GI, and processing degree, modulates sweetness perception, glycemic response, and satiety, thereby shaping obesity risk [55,58]. Additionally, specific fatty acids and amino acids influence appetite and energy balance through hormonal and metabolic pathways [58,59]. Energy homeostasis is maintained through an integrated gut–brain–endocrine axis, which buffers short-term energy fluctuations [81]. Variability in plasma glucose strongly correlates with appetite regulation and long-term body weight trends [82]. Amino acids also modulate satiety-related hormones and diet-induced thermogenesis, further contributing to appetite control [59]. The discovery of leptin established the endocrine link between adipose tissue and hypothalamic centers, supporting models that describe body weight as the outcome of complex neuroendocrine feedback loops [57]. The modern “fat-stat” hypothesis extends this view, suggesting a genetically determined set point for adiposity maintained through homeostatic feedback [60].

In obesogenic environments, these physiological controls are overridden by highly processed, energy-dense foods, which activate reward-related neural circuits and blunt satiety responses [83,84,85,86]. Dietary fats and sugars, particularly endocannabinoid precursors and rapidly absorbed carbohydrates, modulate hedonic eating and reward sensitivity [87,88,89]. Furthermore, circadian misalignment and sleep deprivation disrupt glucose and lipid homeostasis, amplifying the risk of weight gain and metabolic dysfunction [61,90,91].

Metabolic flexibility, the ability to alternate between glucose and fat oxidation, is a critical determinant of energy balance [62]. Regular physical activity enhances this flexibility, improving fat oxidation and insulin sensitivity in individuals with obesity [92]. Similarly, gut microbiota composition and bile acid signaling influence energy extraction, appetite regulation, and interindividual responses to diet [63,64,93]. Overall, current evidence suggests that diet quality, particularly carbohydrate and fat type, exerts a stronger influence on body weight than total macronutrient proportion [94]. Diets rich in whole grains, low-GI carbohydrates, and unsaturated fats are protective, whereas refined carbohydrates, sugar-sweetened beverages, and saturated fats are associated with greater obesity risk [95].

Adequate protein intake supports satiety and energy expenditure, although long-term data indicate that excessive animal protein may favor weight gain, whereas plant-based proteins have protective effects [96]. Thus, both macronutrient distribution and nutrient quality are pivotal for sustainable weight regulation.

## 5. Specific Macronutrient Deficiencies in Obesity

### 5.1. Protein

Protein requirements in obese children are frequently underestimated, particularly during caloric restriction [97]. For children aged 4–18 years, the American Academy of Family Physicians and the American Academy of Pediatrics recommend that protein should constitute 10–30% of total energy intake [98]. Insufficient protein intake accelerates lean tissue loss and contributes to sarcopenic obesity, characterized by reduced skeletal muscle mass coexisting with excess adiposity [99]. This phenotype worsens insulin resistance, physical performance, and overall metabolic health. Chronic low-grade inflammation, an established hallmark of obesity, impairs anabolic signaling and reduces protein utilization efficiency [100]. Adequate protein intake is therefore essential to preserve lean mass and metabolic function during weight management.

Protein quality and source are equally critical. Lean animal proteins (e.g., poultry, eggs, dairy) enhance satiety and thermogenesis, whereas excessive red and processed meat intake increases cardiometabolic risk. Conversely, plant-based proteins from legumes, soy, and nuts provide essential amino acids along with fiber, unsaturated fats, and bioactive compounds that reduce inflammation [101]. A plant-forward protein strategy thus preserves lean mass while mitigating obesity-related inflammatory and metabolic derangements.

### 5.2. Carbohydrates

Carbohydrates should provide 45–65% of total energy intake, and Carbohydrate quality is a primary determinant of obesity risk [98]. Diets in obese children are often dominated by refined starches and sugar-sweetened beverages [102]. These high–GI foods trigger rapid glucose surges and hyperinsulinemia, promoting fat storage and insulin resistance [103]. Chronic consumption of refined carbohydrates contributes to metabolic syndrome and type 2 diabetes [104]. In contrast, complex carbohydrates from whole grains, legumes, fruits, and vegetables blunt glycemic variability, improve satiety, and support a favorable gut microbiota profile [105]. Resistant starches and slowly digestible carbohydrates yield short-chain fatty acids (SCFAs) that strengthen intestinal barrier function and enhance insulin sensitivity. Carbohydrate diversity fosters microbial richness, whereas monotonous refined-carbohydrate diets promote dysbiosis. A chrononutritional dimension is increasingly recognized: evening consumption of high-GI carbohydrates worsens nocturnal glycemic control and lipid metabolism, while daytime intake of low-GI foods aligns with circadian metabolic rhythms. Thus, both carbohydrate quality and timing critically influence obesity development.

### 5.3. Fat

Fat should provide 45–65% of total energy intake, and the quality of dietary fat plays a decisive role in obesity-related metabolic outcomes [98].

Excessive intake of saturated fats, particularly from processed and fried foods, is linked to systemic inflammation, endothelial dysfunction, and hepatic steatosis [43]. Conversely, inadequate consumption of omega-3 polyunsaturated fatty acids (PUFAs) contributes to adverse adipokine profiles and reduced insulin sensitivity [106]. Optimizing fat quality requires both limiting saturated and trans fats and increasing unsaturated fats from nuts, seeds, avocados, and fish oils [107]. Macronutrient interactions further influence lipid metabolism. High-fat, low-fiber diets aggravate endotoxemia and gut-derived inflammation through altered bile acid signaling. In contrast, diets rich in mono- and polyunsaturated fats combined with sufficient fiber improve lipid handling and cardiometabolic outcomes [108]. The timing of fat intake also affects metabolic health: excessive evening fat consumption heightens postprandial triglyceride excursions and worsens insulin sensitivity [109]. In pediatric populations, early exposure to high saturated fat intake is associated with endothelial dysfunction, while insufficient omega-3 intake impairs neurocognitive development. Thus, early dietary correction of fat quality is fundamental to preventing long-term obesity complications.

### 5.4. Fiber

Although classified as part of carbohydrates, dietary fiber merits distinct consideration due to its critical role in metabolic and gut health. Fiber intake should be at least 25 g/day for children older than 4 years [98], but fiber intake among obese children consistently falls short of recommendations [105], contributing to increased energy density, reduced satiety, and impaired glycemic regulation. Fermentable fibers (e.g., inulin, pectins, resistant starches) are metabolized by gut microbiota into SCFAs, acetate, propionate, and butyrate, that regulate hepatic glucose output, lipid metabolism, and inflammation. These metabolites also enhance intestinal barrier function and promote microbial diversity, creating an anti-inflammatory gut milieu. Insoluble fibers, while less fermentable, improve satiety and bowel function, complementing the metabolic benefits of soluble fibers [110]. Longitudinal pediatric studies demonstrate that higher fiber intake correlates with lower BMI, improved lipid profiles, and better insulin sensitivity. Furthermore, fiber-rich diets displace energy-dense, nutrient-poor foods, improving overall nutrient adequacy. Educational and experiential interventions, such as school-based cooking or gardening programs, have proven effective in fostering long-term fiber intake habits and promoting metabolic health in children [111].

The interplay among different macronutrients contributes substantially to the pathophysiology of obesity-related malnutrition. Table 5 summarizes the key biochemical mechanisms, clinical consequences, and dietary strategies associated with each macronutrient class.

## 6. Specific Micronutrient Deficiencies in Obesity

### 6.1. Vitamin D Deficiency

Vitamin D deficiency is highly prevalent among individuals with obesity, affecting 57–94% of this population [114]. This broad range itself highlights methodological heterogeneity in defining deficiency and in population sampling. Contributing factors include poor dietary intake, limited sunlight exposure, and sequestration of vitamin D in adipose tissue, which lowers its bioavailability [115]. Serum vitamin D levels are inversely correlated with BMI, with greater adiposity amplifying deficiency [116]. The theory of volumetric dilution has been proposed as an alternative explanation to sequestration, illustrating that the underlying mechanism is not fully resolved. Furthermore, while numerous cross-sectional studies show an association between low vitamin D and obesity, evidence from supplementation trials for weight loss or major metabolic improvements has been largely inconsistent, suggesting the deficiency may be a marker rather than a direct mediator of metabolic dysfunction in many cases [41]. Vitamin D regulates adipogenesis, inhibits adipocyte proliferation, and enhances insulin sensitivity through GLUT4-mediated glucose uptake. Deficiency is associated with insulin resistance, type 2 diabetes, and sarcopenia [117]. It also impairs calcium absorption and bone mineralization [118] and contributes to muscle weakness and atrophy [119]. Thus, obesity-related vitamin D deficiency perpetuates metabolic dysfunction and musculoskeletal deterioration, reinforcing the pathological cycle of obesity [41].

The high prevalence of deficiency warrants systematic screening with serum 25-OH Vitamin D in all children with obesity. Levels below 20 ng/mL (50 nmol/L) are diagnostic of deficiency and indicate a need for supplementation, with dosing guided by the degree of deficiency and age-specific guidelines, alongside advice on safe sun exposure.

### 6.2. Calcium Deficiency

Calcium, primarily obtained from dairy products and to a lesser extent from leafy greens and fortified foods, plays a pleiotropic role in metabolic regulation. It influences adipocyte function, energy expenditure and muscle integrity. Calcium modulates adipogenesis, lipid metabolism, and adipocyte survival/proliferation, with low intake potentially promoting fat accumulation [120]. Dietary calcium enhances brown adipose tissue (BAT) thermogenesisvia UCP1 activation, suggesting a protective role against obesity [121]. As a critical regulator of muscle contraction and signaling, chronic hypocalcemia may accelerate sarcopenia and muscle wasting [122]. Calcium intake should be 700 mg/day for children aged 1–3 years and 1000 mg/day for those aged 4–8 years. In obesity, low calcium intake, often due to poor dietary quality, may exacerbate metabolic dysfunction while increasing susceptibility to musculoskeletal decline.

### 6.3. Iron Deficiency

Iron deficiency is a common but underrecognized consequence of obesity, primarily mediated by chronic inflammation and elevated hepcidin levels [123]. Hepcidin degrades ferroportin, the key transporter responsible for intestinal iron absorption, leading to functional iron deficiency even when dietary intake is sufficient [124]. This condition impairs oxygen transport, mitochondrial function, and immune responses, compounding metabolic dysfunction [125]. Women of reproductive age are particularly vulnerable due to menstrual losses. Co-ingestion of vitamin C can enhance absorption, yet persistent inflammation limits efficacy. Targeted management of hepcidin-mediated iron dysregulation remains a clinical priority in obesity care.

Iron status should be assessed using ferritin in conjunction with a complete blood count. In the context of obesity and elevated CRP, a low ferritin (<30 µg/L) indicates functional iron deficiency requiring oral iron supplementation. Monitoring hepcidin levels remains a research tool.

### 6.4. Magnesium Deficiency

Magnesium is fundamental to glucose metabolism and insulin action, and deficiency is frequent in obesity due to inadequate intake and increased renal excretion [126]. Low magnesium status contributes to insulin resistance, oxidative stress, and inflammation [127]. As a cofactor for thiamine diphosphate, magnesium supports carbohydrate metabolism; high-sugar diets deplete both nutrients, diverting metabolism toward lipogenesis and exacerbating adiposity [128]. For magnesium, studies in US children aged 4–8 years indicate that an intake of approximately 110–130 mg/day estimated average requirement (EAR) and 130–150 mg/day recommended dietary allowance (RDA) is appropriate, with net retention supporting normal growth and bone health [129,130]. For younger children (1–3 years), the EAR is about 65 mg/day and the RDA is 80 mg/day [130].

Adequate intake from vegetables, legumes, nuts, and seeds is therefore essential for maintaining metabolic integrity and mitigating insulin resistance in obesity.

### 6.5. Zinc Deficiency

Zinc is critical for immune function, antioxidant defense, and insulin signaling, yet obesity is frequently associated with low serum zinc due to poor dietary intake and altered zinc homeostasis [131]. For zinc, the EAR for children aged 1–3 years is 2.5 mg/day and the RDA is 3 mg/day; for children aged 4–8 years, the EAR is 4 mg/day and the RDA is 5 mg/day [132,133].

Deficiency disrupts insulin sensitivity and enhances inflammatory signaling, exacerbating metabolic derangements typical of obesity [131].

### 6.6. B Vitamins

B vitamins, particularly thiamine (B1), folate (B9), and vitamin B12, are indispensable for energy metabolism, methylation, and red blood cell synthesis. Deficiencies are prevalent in obesity due to poor diet quality, increased metabolic demands, and malabsorption after bariatric surgery [134]. Thiamine (B1) acts as a coenzyme for transketolase and the pyruvate dehydrogenase complex. Deficiency impairs glucose oxidation, increases lactate accumulation, and worsens insulin resistance [34]. High-sugar diets accelerate thiamine depletion, compounding hyperglycemia risk [40]. Folate (B9) and vitamin B12, crucial for DNA synthesis and methylation, are often suboptimal in obesity. Vitamin B12 deficiency, common in small intestinal bacterial overgrowth (SIBO) due to bacterial competition, impairs lipid metabolism, protein synthesis, and neuromuscular function [122,135,136].

Collectively, B-vitamin deficiencies worsen insulin resistance, dyslipidemia, and cardiovascular risk. Ensuring adequate intake through diet, monitoring post-surgical status, and targeted supplementation are essential strategies to improve metabolic outcomes in obesity.

### 6.7. Synthesis: The Nutritional Quality Deficit in Obesity

The analysis of macro- and micronutrient status reveals a consistent and critical theme: pediatric obesity is characterized by a profound qualitative nutrient deficit coexisting with energy surplus. The common dietary pattern, excessive saturated fats and refined carbohydrates coupled with inadequate fiber, high-quality protein, and essential vitamins and minerals, creates a perfect storm for metabolic dysfunction. The deficiencies in vitamin D, iron, magnesium, and zinc are not merely incidental findings but active contributors to insulin resistance, inflammation, and oxidative stress. This evidence underscores that the clinical assessment of a child with obesity must extend beyond caloric intake and BMI to include a critical evaluation of dietary quality and micronutrient status, redefining malnutrition in this context as a failure to meet nutritional quality requirements.

## 7. Advances in Biomarker Research

Recent progress in metabolomics and proteomics has substantially improved the early detection of malnutrition by identifying molecular signatures before clinical manifestation. Alterations in branched-chain and aromatic amino acids have been linked to malnutrition in chronic diseases, offering potential avenues for personalized intervention [137]. Digital biomarkers, including wearable sensors that monitor dietary intake, energy expenditure, and physical activity, are emerging as complementary tools to traditional assays, providing real-time insights into nutritional status [138]. Despite their promise, issues related to data privacy, standardization, and user adherence must be addressed before widespread clinical integration.

It is crucial to distinguish between research biomarkers and those ready for clinical application. The following subsections discuss a spectrum of biomarkers, but clinicians should focus on those that are readily available, standardized, and have clear interpretive guidelines (e.g., Vitamin D, ferritin). Other biomarkers, while mechanistically insightful, currently lack the standardization and evidence base to guide individual patient management.

### 7.1. Genetic Damage

Telomere-associated DNA damage is a recognized consequence of metabolic dysregulation in obesity. Studies in children and adolescents demonstrate a strong association between obesity and accelerated telomere shortening, reported in 85.7% of studies [139,140,141,142]. Encouragingly, weight loss interventions have been shown to promote telomere elongation, suggesting partial reversibility of obesity-related genomic damage [143,144]. Improvements in glucose tolerance following dietary modification correlate with increased telomere length, reinforcing the link between metabolic health and genomic stability [145,146]. These findings underscore that telomere dynamics reflect metabolic stress, and restoring metabolic balance may mitigate obesity-induced genetic injury.

### 7.2. Adipose Tissue Biomarkers

Adipose tissue dysfunction is central to obesity-related metabolic disease. Hypertrophic adipocytes alter endocrine function, disrupting adipokine secretion, particularly leptin and adiponectin [147].

Leptin, a key regulator of energy homeostasis, is elevated in obesity and correlates strongly with insulin resistance [148]. In contrast, caloric restriction reduces leptin levels, signaling energy deficit to the hypothalamus [149]. Adiponectin exerts anti-inflammatory and insulin-sensitizing effects, yet its circulating concentrations are typically reduced in obesity, further promoting metabolic dysfunction [150]. The leptin–adiponectin imbalance thus represents a key biomarker axis of metabolic stress and adipose tissue pathology).

### 7.3. Liver Biomarkers

Human serum albumin (HSA) and prealbumin are well-established indicators of nutritional and hepatic function. In obesity, mild hypoalbuminemia is common and becomes more pronounced with the onset of type 2 diabetes and metabolic syndrome [151,152]. Additionally, an elevated urinary albumin-to-creatinine ratio (UACR) signals early renal involvement secondary to metabolic dysfunction [153]. These markers together reflect the systemic metabolic burden of obesity and its impact on protein metabolism and renal health.

### 7.4. Pro-Inflammatory Cytokine Biomarkers

Chronic low-grade inflammation is a defining feature of obesity, driven by dysregulated cytokine signaling. Tumor necrosis factor-alpha (TNF-α) impairs glucose uptake in adipose and muscle tissues through MAPK and IKK pathway activation, directly contributing to insulin resistance [154]. Elevated interleukin-6 (IL-6) and reduced interleukin-10 (IL-10) exacerbate systemic inflammation, while weight loss significantly reduces circulating cytokine levels [154]. This state of chronic, low-grade inflammation, characterized by elevated levels of pro-inflammatory cytokines such as TNF-α and IL-6, may also explain the significantly increased susceptibility to severe outcomes from infections like COVID-19 observed in individuals with obesity [155,156].

It is hypothesized that the pre-existing inflammatory state in obesity creates a heightened baseline, making these individuals more vulnerable to this catastrophic immune response, thereby increasing the risk of severe respiratory distress, multi-organ failure, and death. This illustrates a direct pathophysiological link between nutritional status, adipose-tissue-driven inflammation, and impaired immune competence. When individuals with obesity contract COVID-19, the SARS-CoV-2 virus can trigger a cytokine storm, an excessive and dysregulated release of cytokines, which is a hallmark of severe COVID-19 and is directly linked to multiorgan failure and increased mortality [155,157,158,159].

The chronic inflammation and immune dysregulation in obesity—mediated by mechanisms such as NLRP3 inflammasome activation, impaired interferon responses, and increased expression of viral entry receptors in adipose tissue—amplify the risk and severity of this cytokine storm [160,161,162,163,164,165].

Overall, while direct measurement of TNF-α or IL-6 is not routine, the readily available high-sensitivity CRP (hs-CRP) serves as a practical clinical proxy for this systemic inflammation. An elevated hs-CRP in an obese child can reinforce the diagnosis of a high-inflammatory metabolic phenotype and underscore the importance of interventions aimed at reducing inflammatory drivers, primarily through improved diet quality and weight management.

## 8. Effect of Gut Microbiota on Obesity

### 8.1. Early-Life Determinants and Individual Variability

The gut microbiota is shaped throughout life by diverse environmental and developmental factors, mode of delivery, early feeding practices, antibiotic exposure [166], and habitual diet. These determinants, analogous to genetic influences, modulate early microbial colonization and community composition [167], supporting a personalized medicine approach to obesity. However, it is crucial to distinguish between compelling associations and proven causality, a challenge that pervades much of the microbiome literature. Some authors consider the microbiota a key environmental determinant closely integrated with host metabolism [168]. However, whether microbiota alterations are a cause or consequence of obesity remains uncertain. Dietary macronutrients, especially fiber-rich carbohydrates, proteins, and fats, exert consistent, though individualized, effects on microbial ecology [169]. Despite shared dietary influences, microbiota composition remains highly individualized, reflecting both metabolic status and body weight [169].

### 8.2. Microbial Signatures of Obesity

Pioneering studies, primarily in animal models, identified characteristic microbial shifts in obesity, notably an increased *Firmicutes*-to-*Bacteroidetes* ratio [170]. Similar trends have been observed in humans, where obese phenotypes exhibit reduced microbial diversity, increased Prevotellaceae and Archaea, and greater energy harvest from food [171]. However, subsequent human studies have revealed significant inconsistency, with many failing to replicate this specific finding [172]. Low microbial richness correlates with insulin resistance, dyslipidemia, and systemic inflammation [173], while specific taxa such as *Akkermansia muciniphila* appear protective by modulating the endocannabinoid system and energy balance [174]. Variability across studies reflects the influence of host genetics [175], sex, and dietary composition [176]. The enterotype concept, distinct clusters of microbial communities, helps explain interindividual metabolic diversity [177].

Metabolically, microbial activity centers on SCFAs, which increase energy yield from indigestible substrates [178] and regulate host thermogenesis via PPARG and UCP2, enhancing lipid oxidation [179]. SCFAs also influence appetite regulation, as colonic infusion increases peptide YY (PYY) through GPR41/GPR43 signaling [180]. However, correlations between fecal SCFA levels and bacterial composition remain inconclusive; further intervention studies are required to clarify their causal role in weight regulation.

Dietary macronutrients remain major modulators of microbial ecology. Dietary fiber shapes microbial composition, intestinal transit, and satiety responses [181,182]. Fermentable fibers promote SCFA synthesis and microbial diversity [183]. Prebiotics such as fructo-oligosaccharides enhance *Faecalibacterium prausnitzii* and butyrate production, reducing appetite via hypothalamic neuropeptide Y downregulation [184].

In contrast, Western-style diets, rich in refined sugars and artificial sweeteners, favor Firmicutes proliferation and upregulate glycolytic and gluconeogenic pathways [185]. SIBO has been linked to carbohydrate-rich, fiber-poor diets [186]. Under low-carbohydrate conditions, protein fermentation increases production of branched-chain amino acids and phenylacetic acid, compounds with potentially deleterious metabolic effects [187]. Conversely, tryptophan-derived metabolites exert anti-inflammatory, barrier-strengthening, and appetite-regulating actions via GLP-1 [188]. Dietary fats also influence microbiota composition: high-fat diets induce dysbiosis and low-grade inflammation [189], whereas MUFA and *n*-3 PUFA intake supports Bifidobacterium and Lactobacillus abundance [190,191]. The use of probiotics may offset adverse microbial changes, though clinical findings remain inconsistent [192].

### 8.3. Microbiota Dysfunction in Obesity

Individuals with obesity frequently exhibit microbiota dysfunction that compromises nutrient absorption and metabolic regulation. In clinical studies, low-calorie diets often fail to normalize micronutrient status, suggesting impaired nutrient utilization linked to microbial imbalance [193]. Gut bacteria also contribute to systemic inflammation via lipopolysaccharide (LPS)–TLR4 activation [194], with elevated plasma LPS and intestinal permeability observed in obesity [195]. Animal studies demonstrate causality: germ-free mice colonized with microbiota from obese donors gain more weight than controls despite identical diets, indicating transmissibility of the obese phenotype [171]. The *Firmicutes*/*Bacteroidetes* ratio remains a recurring feature, with *Bacteroidetes* restoration following weight loss [196,197]. The observed trends are highly dependent on methodology (e.g., 16S rRNA vs. shotgun metagenomics), geography, diet, and host genetics. This heterogeneity underscores that a single ‘obese microbiota’ profile is likely an oversimplification, and the relationship is influenced by a multitude of confounding factors.

Pregnancy introduces additional variability, overweight women exhibit elevated *Staphylococcus* and *Bacteroidetes* levels compared to normal-weight women, as measured by both fluorescent in situ hybridization and quantitative PCR techniques [198]. Overweight status in pregnancy correlates with higher concentrations of these taxa, which are linked to reduced microbial diversity and altered metabolic profiles, including increased plasma cholesterol and changes in folic acid and triglyceride levels [199,200].

These microbiota shifts are associated with excessive gestational weight gain and may contribute to maternal complications such as gestational diabetes, hypertensive disorders, and dyslipidemia [201,202] or neonates, maternal overweight and associated microbiota changes, particularly increased Bacteroides, are implicated in the initial assembly of the infant gut microbiome, which may predispose offspring to higher birth weight and increased risk of childhood obesity and metabolic disease [203,204].

The transmission of these microbial patterns is more pronounced following vaginal delivery, and the altered infant microbiome may persist for months, potentially affecting long-term metabolic programming [205].

Nonetheless, results across studies are heterogeneous, partly due to methodological differences in microbiota profiling [206]. Other modifiers include age, sex, genetics, and environment [168]. Therapeutic microbiota modulation remains promising but not yet definitive: probiotic, prebiotic, and antibiotic interventions show variable efficacy, underscoring the need for standardized randomized trials [206]. Emerging advances in microbiome sequencing and personalized nutrition hold potential to refine obesity management and prevention strategies [206,207,208].

### 8.4. Critical Appraisal of Microbiota Evidence

It is crucial to interpret the literature on gut microbiota and obesity with caution. The vast majority of evidence is correlative from observational studies, which cannot establish causality. Whether microbial dysbiosis is a cause or a consequence of obesity remains a key unanswered question [209]. Intervention studies with probiotics or prebiotics have shown variable and often modest effects [210], indicating that therapeutic manipulation of the microbiome is complex and not yet well-established. Future research employing longitudinal designs, standardized analytical methods, and fecal microbiota transplantation studies in humans will be essential to move from association to causation.

## 9. Precision Nutrition, Gut Microbiota, and Malnutrition in Obesity: Integrated Perspectives

Precision nutrition represents a promising frontier for redefining obesity management by integrating genomic variability, microbiota composition, and other individual determinants [113]. However, it is critical to note that this field is still in its nascent stages. While controlled-feeding trials demonstrate the potential for personalized dietary responses [211], the translation of these findings into scalable, effective clinical interventions is limited and largely exploratory. Therefore, the current clinical approach must prioritize foundational, evidence-based strategies (dietary quality improvement, physical activity, and targeted micronutrient repletion) while recognizing that truly personalized algorithms based on omics data are not yet part of standard care. Most models lack robust external validation, and the cost-effectiveness of widespread multi-omics profiling for dietary advice has not been established. Furthermore, the effect sizes of genetic or microbial predictors are often small compared to the overwhelming impact of fundamental socioeconomic and environmental factors. Therefore, while precision nutrition holds great future potential, its current application in routine pediatric obesity care remains limited. Long-term patterns exert lasting influence: *Bacteroidetes* predominate in protein- and fat-rich diets, whereas *Prevotella* aligns with carbohydrate-based patterns [211]. In rodents, high-fat/high-sugar diets reduce microbial diversity, induce intestinal inflammation, and disrupt gut–brain signaling, thereby promoting adiposity [212].

Integrative microbiome–metabolome profiling now enables objective monitoring of adherence to healthy dietary patterns, such as the Mediterranean diet, and their metabolic correlates [213]. In obesity, adherence to nutrient-dense diets correlates with higher microbial richness and lower systemic inflammation [214]. Nonetheless, the causal relationships between diet-induced microbial changes and metabolic improvement require further investigation [215]. Emerging evidence also links host genotype–microbiota interactions with obesity risk [216]. Individuals carrying Bifidobacterium-related LCT variants exhibit improved outcomes under low-calorie, high-protein diets [217]. Hence, macronutrient distribution, microbial ecology, and genetic background jointly shape metabolic responses to diet [218]. Screening tools based on enterotypes, microbial metabolites, and host phenotypic signatures could facilitate individualized weight management [219].

Achieving true precision nutrition will require integrating broader determinants, lifestyle, sociocultural context, medical history, sleep, physical activity, circadian rhythms, epigenetics, and metabolomics, into a unified clinical framework [220]. Energy imbalance remains the core mechanism driving obesity, yet the relative contribution of fats and sugars is difficult to disentangle due to overlapping metabolic pathways [221,222].

Protein enhances satiety and thermogenesis [55], whereas fiber improves satiety and long-term appetite regulation [223]. Specific amino acids, fatty acids, and genetic variants, such as FTO polymorphisms, modulate susceptibility to weight gain, though findings remain inconsistent [222]. Despite dietary guidelines, saturated fat intake continues to exceed AHA recommendations, while omega-3 fatty acid consumption remains insufficient and trans fats, though reduced, still contribute to total energy intake [224]. The Protein Leverage Hypothesis further posits that low dietary protein (<14% of total energy) drives compensatory overeating, aligning with global trends linking reduced protein density to rising obesity prevalence [225].

Obesity often clusters within families. Maternal obesity is a strong predictor of childhood obesity, likely mediated by intrauterine programming and early nutritional exposure [226]. Persistent family dietary patterns amplify these risks. The higher obesity prevalence among women, even under similar environmental conditions, suggests possible sex-specific metabolic susceptibilities. Obesity-related malnutrition represents a paradox of caloric excess and micronutrient deficiency, underpinned by poor diet quality, inflammation, and metabolic stress [227]. Effective management requires multilevel strategies: a) clinical monitoring and targeted supplementation for key deficiencies (vitamin D, iron, magnesium, B vitamins), b) development of standardized biomarker panels combining biochemical, inflammatory, and microbiota-related indices to enhance diagnostic accuracy and c) longitudinal monitoring of micronutrient status to prevent complications such as anemia, osteoporosis, and neuropathy.

Nutrient-dense dietary patterns remain the foundation of therapy, with fortification used where necessary. Precision medicine can further refine care: genetic profiling, microbiome analysis, and metabolic biomarkers enable tailored dietary interventions (e.g., optimizing nutrient bioavailability in insulin-resistant individuals). Ultimately, progress requires coordinated action among clinicians, researchers, policymakers, and the food industry. Expanding access to affordable, high-quality foods, especially in low-income settings, is essential to combat the dual burden of overweight and hidden hunger, and to foster sustainable, equitable obesity prevention worldwide

## 10. Synthesis and Hierarchy of Evidence

This comprehensive review has synthesized a vast body of literature on the complex phenomenon of malnutrition in pediatric obesity. To provide clarity and a practical guide for clinicians and researchers, the evidence is categorized below based on its current level of scientific support.

### 10.1. Consensual and Well-Established Evidence

It is now a consensus that pediatric obesity is characterized by a dual burden of excessive caloric intake and concurrent deficiencies in essential micronutrients such as vitamin D and iron [228]. The primary driver of this condition is the consumption of energy-dense, nutrient-poor diets, high in ultra-processed foods, refined carbohydrates, and saturated fats, while being low in fiber, fruits, and vegetables [229]. This dietary pattern is unequivocally linked to a state of chronic low-grade inflammation and a significantly increased risk for cardiometabolic comorbidities, even in childhood.

### 10.2. Probable and Plausible Mechanisms

A substantial body of evidence makes it highly plausible that systemic inflammation is not merely a consequence but an active mechanism driving nutrient deficiencies, for instance, by elevating hepcidin and impairing iron absorption [230]. The gut microbiota is likely a significant modifier of metabolic health, with dysbiosis contributing to inflammation, energy harvest, and nutrient metabolism. Furthermore, the concept of sarcopenic obesity is increasingly supported as a clinically relevant complication.

### 10.3. Hypothesis-Level and Speculative Concepts

While the gut microbiota is a compelling area of research, the assertion that specific microbial signatures (like the *Firmicutes*/*Bacteroidetes* ratio) are a primary cause of human obesity remains speculative, as human evidence is largely correlative. Consequently, therapeutic strategies targeting the microbiome (specific probiotics, prebiotics) show promise but are not yet consistently effective as standalone treatments. Similarly, the field of precision nutrition, while holding great future potential, is currently limited by a lack of validated, scalable algorithms for routine clinical practice in pediatrics.

### 10.4. Critical Gaps in Pediatric Research

A major limitation in translating these findings to clinical practice is the relative scarcity of high-level evidence specifically from pediatric cohorts. This includes a lack of long-term intervention studies, robust validation of diagnostic criteria for malnutrition in obese children, and a clear understanding of how nutritional needs during critical growth periods interact with the pathophysiology of obesity.

## 11. Limitations of the Research on Malnutrition in Pediatric Obesity

While the previous sections have synthesized the current evidence on the mechanisms and manifestations of malnutrition in pediatric obesity, it is critical to assess how the inherent limitations of the available scientific literature affect the certainty of the conclusions we can draw in this review. The following subsections delineate the major methodological and conceptual gaps in the field and, most importantly, reflect on how these constrain the interpretation of our claims and the generalizability of our clinical and research recommendations.

### 11.1. Inconsistent Prevalence Estimates for Pediatric Malnutrition

Historically, the absence of a standardized definition for pediatric malnutrition, both in developing and developed countries, has resulted in highly variable prevalence estimates (ranging from 6% to 51%) and inconsistent screening practices across institutions [231]. These discrepancies primarily reflect differences in diagnostic criteria, anthropometric indicators, and socioeconomic contexts. Establishing harmonized definitions, such as those aligned with the GLIM or ASPEN criteria, is essential to improve comparability and facilitate robust meta-analyses [24].

### 11.2. Limited Evidence for Diagnostic Accuracy of Malnutrition Criteria

Although consensus-based indicators for pediatric malnutrition have been proposed, evidence regarding their diagnostic accuracy, specifically sensitivity, specificity, and predictive validity, remains limited [232]. Clinicians are encouraged to record quantitative parameters to enable large-scale validation of these indicators. Prospective, multicentre studies comparing diagnostic tools against clinical outcomes are necessary to establish reproducibility and diagnostic reliability. Therefore, our endorsement of the ASPEN/GLIM criteria (in Section 2) is based on expert consensus and face validity rather than robust, prospective validation. Our recommendations for their use in clinical practice should be viewed as a proposed framework for standardizing care, not as an evidence-based gold standard proven to predict clinical outcomes superiorly to other methods. The certainty of our statements regarding diagnosis is therefore moderate and calls for further validation.

### 11.3. Inconclusive Microbiota Modulation for Obesity Treatment

Therapeutic modulation of the gut microbiota remains a promising yet inconclusive field. Studies investigating probiotics, prebiotics, and antibiotics have reported inconsistent outcomes, often limited by small sample sizes, heterogeneous designs, and short intervention durations [233]. Future randomized controlled trials should incorporate standardized microbial endpoints and stratify participants according to baseline microbiota composition to clarify the therapeutic potential of microbiome-targeted strategies.

This directly constrains the practical recommendations we can make in Section 8 and Section 9. While we discuss the potential of microbiota-targeted therapies, our conclusion must be that there is currently insufficient evidence to recommend specific probiotic strains, prebiotics, or synbiotics as a standard, effective treatment for obesity-related malnutrition. Thus, our discussion of therapeutic modulation remains speculative and forward-looking, rather than a core component of current clinical management guidelines.

### 11.4. Inconsistent Evidence for Macronutrient Effects on Weight Gain

Evidence linking specific amino acids, fatty acids, and genetic polymorphisms to weight gain susceptibility remains inconsistent. This uncertainty likely reflects the complex interaction between gene expression, nutrient metabolism, and gut microbial composition. Integrative nutrigenomic and metabolomic approaches are required to elucidate these relationships and define causal pathways.

This inconsistency necessitates caution in our interpretation of the various nutritional models (CIM, EBM, PLH) detailed in Section 4. It suggests that no single model is likely to be universally correct and that the mechanisms linking diet to obesity are highly complex and individualized. Therefore, our synthesis of these models should be seen as an overview of competing, non-mutually exclusive frameworks, and our conclusions favor a balanced, diet-quality-focused approach (as in Section 5) over strong claims for the primacy of any single macronutrient.

### 11.5. Unclear Role of the Microbiome in Obesity Causality

A significant limitation in the field is the predominance of correlative studies, which leaves the causal role of the gut microbiome in obesity unresolved. While animal fecal microbiota transplantation studies suggest causality [234], direct evidence in humans is sparse. Furthermore, as noted in Section 8, the highly publicized *Firmicutes*/*Bacteroidetes* ratio has proven to be an inconsistent biomarker across human populations [172]. This inconsistency largely stems from methodological heterogeneity in sequencing techniques, bioinformatic pipelines, and cohort characteristics. The field urgently requires standardized protocols and reporting frameworks to ensure reproducibility and meaningful comparison across studies.

This fundamental limitation means that, while our review describes plausible associations between dysbiosis, inflammation, and malnutrition (e.g., in Section 4 and Section 8), we cannot state with certainty that microbiota alterations are a primary cause of malnutrition in pediatric obesity, rather than a consequence. Consequently, the mechanistic pathways we outline that involve the microbiota should be interpreted as compelling hypotheses within a theoretical framework, not as established facts. This weakens the certainty of claims that position gut health as a central etiological factor.

### 11.6. Inconsistent Evidence for Probiotics Mitigating Dietary Fat Effects

While probiotics have been proposed to mitigate the adverse metabolic effects of high-fat diets, current evidence remains inconsistent [234]. Outcomes vary depending on strain specificity, dosage, duration, and host-dependent factors. Large, strain-specific clinical trials are necessary to determine efficacy and optimal therapeutic contexts. The heterogeneity of microbial responses to dietary interventions highlights the need for reproducible microbial markers, such as SCFA profiles, α-diversity indices, and inflammatory pathways, that can predict metabolic improvement. Future research should employ longitudinal designs and integrate functional metagenomic data to identify consistent microbial adaptations to nutritional change.

This reinforces the constraint noted in Section 10.3. It specifically tempers any enthusiasm for recommending probiotics as a direct countermeasure to the adverse effects of a high-fat or Western-style diet, a concept that might be inferred from our discussion in Section 8. Our manuscript cannot present this as an effective strategy based on current evidence.

### 11.7. Over-Reliance on Observational Data and Short-Term Interventions

An overarching limitation of the evidence base reviewed is the heavy reliance on cross-sectional and observational studies for establishing associations between diet, nutrients, and obesity. These designs are susceptible to confounding and cannot prove causality. While RCTs exist, they are often of short duration, limiting their ability to assess long-term efficacy and sustainability of dietary interventions, especially in growing children. There is a critical need for more long-term, high-quality RCTs and prospective cohort studies that account for the complex interplay of dietary, behavioral, and environmental factors.

## 12. Conclusions

Pediatric obesity underscores the paradox of energy excess coexisting with nutrient inadequacy.

This review has delineated the path from poor dietary quality to a self-perpetuating cycle of systemic inflammation, gut dysbiosis, and functional micronutrient deficiencies. The link between a diet high in processed foods and a state of ‘hidden hunger’ is well-established, as are the consequent risks for cardiometabolic disease and impaired growth. We have shown it is plausible that inflammation and microbiota alterations are key mechanistic drivers of this paradox. Malnutrition should therefore be defined not only by body size but by the quality and balance of nutrients consumed.

However, the translation of this knowledge into precise clinical tools, such as reliable microbiota modulation or universally applicable precision nutrition algorithms, remains largely speculative and an area for crucial future research. Therefore, effective management requires a dual strategy: precision nutrition tailored to each child’s metabolic and developmental needs, and population-level measures that reshape the food environment. Priorities include reformulating processed foods to improve nutrient density, reducing access to sugar-sweetened beverages and foods rich in saturated fats, and promoting fruits, vegetables, whole grains, and legumes. By integrating individualized care with community- and policy-level interventions, it is possible to mitigate the long-term health consequences of pediatric obesity and foster healthier growth trajectories.

## Figures and Tables

**Table 1:** Forms and determinants of malnutrition in children.

Form of Malnutrition	Definition (WHO Criteria)	Primary Causes and Risk Factors	Major Clinical Consequences	Global Prevalence/Epidemiological Notes
Wasting (acute undernutrition)	BMI-for-age or weight-for-height > 3 SD below WHO median	Acute food deprivation; Infections; poor sanitation.	Rapid weight loss; Muscle wasting; Immune impairment; ⭡ infection risk.	~45 million children < 5 years [23]
Stunting (chronic undernutrition)	Height-for-age > 2 SD below WHO median	Chronic food insecurity; Recurrent infections; Maternal malnutrition; ⭣ birth weight.	Irreversible cognitive impairment; ⭣ adult height and productivity.	~149 million children < 5 years [23]
Underweight	Weight-for-age > 2 SD below WHO median	Inadequate dietary intake; Chronic illness; Socioeconomic deprivation.	Growth retardation; Delayed psychomotor development; ⭡ mortality.	Predominant in low- and middle-income countries [5]
Overweight/Obesity (overnutrition)	Excessive body fat accumulation impairing health	High-energy, nutrient-poor diets; Sedentary lifestyle; genetic predisposition	Insulin resistance, hypertension, dyslipidemia; Early NCD onset.	>340 million children and adolescents globally [1]
Double burden of malnutrition	Coexistence of undernutrition (e.g., stunting) and overweight/obesity within the same population, household, or individual	Rapid urbanization; dietary transition; socioeconomic inequality	Concurrent micronutrient deficiencies, metabolic dysregulation, long-term NCD risk.	Rising prevalence in LMICs (e.g., Malaysia, Zimbabwe) [4]

⭡ increase, ⭣ reduce.

**Table 2:** Mechanistic and nutritional models underlying pediatric obesity.

Model/Hypothesis	Core Mechanism	Principal Dietary Driver	Clinical and Metabolic Implications	Limitations/Ongoing Debates
Carbohydrate–Insulin Model (CIM) [20]	High-glycemic carbohydrates ⭡ insulin secretion, promoting fat deposition, ⭡ appetite.	Refined carbohydrates; added sugars.	Suggests benefit of ⭣-glycemic diets in weight control.	Evidence mixed; Total energy balance remains determinant.
Energy Balance Model (EBM) [21]	Obesity results from sustained positive energy balance (energy intake > expenditure).	Energy-dense processed foods.	Supports calorie ⭣ and physical activity as core interventions.	Oversimplifies hormonal and metabolic adaptation mechanisms.
Fructose Survival Hypothesis (FSH) [20]	Fructose metabolism induces ATP depletion, uric acid accumulation, and fat storage (“survival mode”).	High-fructose corn syrup; sugary beverages.	Links fructose overconsumption to metabolic syndrome and fatty liver.	Limited causal data in children.
Protein Leverage Hypothesis (PLH) [21]	Humans regulate protein intake; when dietary protein is diluted, compensatory overeating of fats/carbs occurs.	Ultra-processed foods ⭣ in protein	Highlights importance of protein density and quality in diet.	Theoretical model; Needs validation in children.
Inflammation–Sarcopenia Axis [19]	Chronic adipose inflammation (TNF-α, IL-6) promotes muscle catabolism (sarcopenic obesity).	Diets ⭡ in saturated fat; ⭣ in micronutrients	Links chronic inflammation to muscle ⭣ and metabolic dysfunction	Poorly characterized in children.

⭡ increase, ⭣ reduce.

**Table 3:** Clinical consequences of obesity-related malnutrition.

Affected System/Function	Underlying Mechanism	Clinical Manifestation	Key Micronutrient(s) Involved
Skeletal (bone health) [19]	Preferential nutrient allocation (vitamin K used for hepatic coagulation rather than bone carboxylation)	⭣ bone mineral density; ⭡ fracture risk.	Vitamin K; Vitamin D; Calcium.
Metabolic/endocrine [38]	Impaired glucose and insulin metabolism due to micronutrient deficiency	Insulin resistance; impaired β-cell function. type 2 diabetes.	Vitamin D; Magnesium. Zinc.
Musculoskeletal [41]	Chronic inflammation; ⭣ protein synthesis leading to loss of lean mass	Sarcopenic obesity; muscle weakness; ⭣ functionality.	Protein; Vitamin D. Magnesium.
Neurological/cognitive [42]	Oxidative stress; Neuroinflammation; micronutrient deficits (B vitamins, antioxidants)	Cognitive impairment; memory decline.	Thiamine; Folate. Vitamin B12; Zinc.
Reproductive (female health) [42]	Hormonal imbalance; Oxidative stress; Micronutrient insufficiency.	Infertility; Menstrual irregularities; Adverse pregnancy outcomes.	Zinc; Iron. Vitamin E.
Cardiovascular [43]	Endothelial dysfunction; Dyslipidemia; Pro-inflammatory cytokine release.	Hypertension; Atherosclerosis; Cardiovascular disease	Vitamin D; Magnesium; Omega-3 fatty acids.
Pediatric population [44]	Combined dietary deficiency; Obesity-related inflammation.	Dyslipidemia; Insulin resistance; Early-onset hypertension.	Iron; Vitamin D. Calcium.
Critical care/systemic [34]	Malnutrition impairs immune and metabolic response during stress.	Poor ICU outcomes. delayed recovery. ⭡ mortality.	Multiple micronutrient deficiencies.

⭡ increase, ⭣ reduce.

**Table 4:** Mechanisms and regulatory pathways involved in energy homeostasis and obesity.

Regulatory Mechanism/Pathway	Core principle or Function	Key Mediators/Components	Effect on Appetite, Metabolism, or Adiposity
Glucostatic theory	Hunger and satiety regulated by glucose availability in the brain (short-term control)	Plasma glucose; hypothalamic glucose sensors.	Low glucose ⭡ hunger; Stable glucose promotes satiety
Lipostatic mechanism [55]	Long-term regulation of body fat through lipid feedback	Adipose tissue; lipid-derived signals;	Maintains stable body fat mass over time.
Carbohydrate quality [56]	Influences sweetness perception, glycemic response, and satiety	Fiber; glycemic index; processing level.	High-quality carbs improve satiety and glycemic control; Refined carbs ⭡ obesity risk.
Gut–neuroendocrine axis [57]	Coordinates short-term energy balance and appetite regulation	Gut peptides; Enteric nervous system.	Buffers energy fluctuations; Influences meal size and frequency.
Amino acid–hormone interactions [58,59]	Amino acids regulate satiety and thermogenesis	Leucine; GLP-1. Other satiety hormones.	Enhance diet-induced thermogenesis; Suppress appetite.
Leptin signaling [57]	Endocrine link between adipose tissue and hypothalamic centers	Leptin; Hypothalamus.	Promotes satiety; Inhibits feeding; Integrates fat mass feedback.
“Fat-stat” hypothesis [60]	Genetic set point for adiposity maintained by feedback mechanisms	Adipose-derived signals; hypothalamic regulation	Stabilizes body fat around a genetically determined set point.
Reward pathway dysregulation [49]	Hyperpalatable; energy-dense foods override homeostatic control	Dopamine; endocannabinoids; sugar–fat combinations	Promotes overeating and food addiction-like behavior.
Circadian regulation [61]	Synchronizes metabolism with sleep–wake cycles	Clock genes; Melatonin; Cortisol.	Circadian misalignment disrupts glucose and lipid metabolism; Promoting weight gain.
Metabolic flexibility [62]	Ability to switch between glucose and fat oxidation	Mitochondrial enzymes; Insulin sensitivity.	Reduced flexibility favors fat storage and metabolic inflexibility.
Gut microbiota and bile acid signaling [63,64]	Microbiota modulate energy extraction and appetite	Bile acids; SCFAs; Gut–brain axis	Dysbiosis alters nutrient absorption and energy balance.
Diet quality and macronutrient source	Carbohydrate and fat type exert stronger effects than total macronutrient amount	Whole grains; Unsaturated fats vs. refined carbs; Saturated fats	High-quality foods protect; refined sugars and fats ⭡ obesity risk.

⭡ increase, ⭣ reduce.

**Table 5:** Macronutrient Quality and Obesity-Related Malnutrition in Children.

Macronutrient	Key Mechanisms	Clinical and Metabolic Impact	Dietary Recommendations/Preventive Strategies
Protein [97,98,99,100,101]	Inadequate intake during caloric restriction promotes lean mass ⭣ and sarcopenic obesity; Chronic inflammation impairs anabolic signaling and protein utilization.	Sarcopenic obesity; ⭡ insulin resistance; Impaired physical function; ⭣ metabolic efficiency.	Ensure adequate protein intake during weight management; emphasize lean animal sources (poultry, eggs, dairy) and plant-based proteins (legumes, soy, nuts) to preserve lean mass and reduce inflammation.
Carbohydrates [98,102,104]	High-GI refined carbs induce postprandial hyperglycemia and hyperinsulinemia; Refined starches and sugars promote insulin resistance and dysbiosis.	⭡ adiposity; ⭡ insulin resistance; ⭡ metabolic syndrome; ⭡ type 2 diabetes.	Prioritize complex, fiber-rich carbohydrates (legumes, whole grains, fruits, vegetables); Diversify carbohydrate sources to enhance microbiota health; Avoid evening high-GI intake to align with circadian metabolism.
Fat [98,106,112,113]	Excess saturated fats promote inflammation, endothelial dysfunction, and hepatic steatosis; ⭣ omega-3 intake worsens adipokine profile and insulin sensitivity; Timing of intake affects lipid metabolism.	Systemic inflammation; Dyslipidemia; Hepatic steatosis; Impaired glucose tolerance; Neurocognitive deficits in children.	⭣ saturated/trans fats; ⭡ mono- and polyunsaturated fats from nuts, seeds, avocados, and fish oil; Distribute fat intake evenly across meals; Ensure adequate omega-3 intake during growth.
Fiber [98,105]	⭣ intake reduces satiety, impairs glycemic regulation, and promotes dysbiosis; Fermentable fibers generate SCFAs that regulate glucose and lipid metabolism.	⭡ risk of weight gain, Insulin resistance; Systemic inflammation; Poor gut health.	⭡ fermentable and insoluble fiber intake through whole grains, fruits, legumes, and vegetables; ⭡ fiber education and experiential nutrition programs in schools.

⭡ increase, ⭣ reduce.

## Data Availability

No new data were created or analyzed in this study. Data sharing is not applicable to this article.

## Abbreviations

The following abbreviations are used in this manuscript:

AHA	American Heart Association
ASPEN	American Society for Parenteral and Enteral Nutrition
BAT	Brown Adipose Tissue
BCAA	Branched-Chain Amino Acids
BMI	Body Mass Index
CD	Celiac Disease
CDC	Centers for Disease Control and Prevention
CIM	Carbohydrate–Insulin Model
EAR	Estimated Average Requirement
DGA	Dietary Guidelines for American
DNA	Deoxyribonucleic Acid
EBM	Energy Balance Model
FHO	Fructose Survival Hypothesis
GLIM	Global Leadership Initiative on Malnutrition
GLP-1	Glucagon-Like Peptide 1
GI	Glycemic Index
GLUT4	Glucose Transporter Type 4
HEI	Healthy Eating Index
HFCS	High-Fructose Corn Syrup
hs-CRP	High-Sensitivity CRP
ICU	Intensive Care Unit
IKK	IκB Kinase
IL-1 β	Interleukin 1 Beta
IL-6	Interleukin 6
IL-10	Interleukin 10
LMICs	Low- and Middle-Income Countries
LPS	Lipopolysaccharide
MAPK	Mitogen-Activated Protein Kinase
MUAC	Mid-Upper Arm Circumference
MUFA	Monounsaturated Fatty Acids
NCD	Non-Communicable Disease
NGF	Nutritional Geometry Framework
NHANES	National Health and Nutrition Examination Survey
PLH	Protein Leverage Hypothesis
PPARG	Peroxisome Proliferator-Activated Receptor Gamma
PUFA	Polyunsaturated Fatty Acids
PYMS	Pediatric Yorkhill Malnutrition Score
RBC	Red Blood Cells
RDA	Recommended Dietary Allowance
SIBO	Small Intestinal Bacterial Overgrowth
SCFA	Short-Chain Fatty Acid
STAMP	Screening Tool for the Assessment of Malnutrition in Pediatrics
STRONGkids	Screening Tool for Risk on Nutritional Status and Growth
TNF-α	Tumor Necrosis Factor Alpha
TLR4	Toll-Like Receptor 4
UACR	Urinary Albumin-to-Creatinine Ratio
UCP1	Uncoupling Protein 1
UCP2	Uncoupling Protein 2
WHO	World Health Organization
